# Effect of Polygain™ Supplementation on Growth Performance, Lesion Severity, and Oocyst Shedding in *Eimeria*-Challenged Broiler Chickens

**DOI:** 10.3390/ani15213130

**Published:** 2025-10-29

**Authors:** Thalia Marina Llalla Vidal, Siraprapa Boobphahom, Suttitas Tongkamsai, Matthew Flavel

**Affiliations:** 1 The Product Makers, Melbourne, VIC 3173, Australia; tvidal@tpm.com.au; 2 Research Centre for Neuroscience, Institute of Molecular Biosciences, Mahidol University, Nakhon Pathom 73170, Thailand; siraprapa.boo@mahidol.ac.th; 3 Department of Veterinary Medicine, Faculty of Veterinary Science, Rajamangala University of Technology Tawan-Ok, Chonburi 20110, Thailand; 4 Department of Microbiology, Anatomy, Physiology and Pharmacology, La Trobe University, Melbourne, VIC 3083, Australia

**Keywords:** anticoccidial, sugar cane polyphenols, broilers

## Abstract

**Simple Summary:**

Coccidiosis is a serious intestinal disease in chickens caused by *Eimeria* parasites that can negatively impact animal welfare and reduce poultry production efficiency. This study tested the effectiveness of Polygain™, a natural supplement made from sugarcane, in helping broiler chickens recover from a multiple *Eimeria* spp. infection. Birds were divided into groups that either received no treatment, a standard anticoccidial drug, or Polygain™ at three different doses. Their growth, gut health, and coccidia shedding levels were measured. The results showed that birds fed Polygain™ had better survival, lower parasite counts, and fewer intestinal injuries compared to untreated birds. The highest dose of Polygain™ even resulted in total remission of the lesion score in the caecum. Polygain™ proved to be a safe, sustainable, and promising alternative in poultry farming systems that aim to reduce reliance on synthetic drugs while maintaining bird health and performance.

**Abstract:**

Coccidiosis, caused by *Eimeria* spp., is a major economic burden in poultry production, prompting growing interest in natural alternatives to synthetic anticoccidials. This study evaluated the use of Polygain™, a sugarcane-derived polyphenol-rich feed material, as a natural anticoccidial in broiler chickens experimentally challenged with *Eimeria tenella*, *E. maxima*, and *E. acervulina*. A total of 144 Ross 308 chicks were allocated to six groups: uninfected–untreated control, infected–untreated control, infected plus nicarbazin + narasin, and three Polygain™ treatment groups (250, 500, and 1000 ppm). Birds were orally challenged with mixed oocysts on day 14. Parameters assessed included body weight gain (BWG), lesion scores (LS), oocyst per gram (OPG), and calculated indices such as relative oocyst production (ROP), reduction in lesion score (RLS), percent optimum anticoccidial activity (POAA), and anticoccidial index (ACI). Polygain™ reduced intestinal lesions (*p* < 0.05), particularly in the caecum, with complete lesion resolution observed at 1000 ppm by 21 days post-infection. Polygain™ ACI values (143–146) were limited in comparison to nicarbazin + narasin treatment (ACI 160). These findings demonstrate that Polygain™ supplementation confers measurable protective dose related effects against coccidiosis without impairing growth performance, supporting its potential as a natural feed material for integrated coccidiosis management.

## 1. Introduction

Coccidiosis, caused by multiple *Eimeria* infections in chickens, is recognized as a major issue in the poultry industry worldwide. This condition results in significant economic losses, with a global cost estimated at £10.4 billion, which equates to £0.16 per chicken [1].

Among the seven *Eimeria* species infecting chickens, *E. acervulina*, *E. maxima*, and *E. tenella* account for most clinical cases [2]. Each species targets specific regions of the gastrointestinal tracts: *E. acervulina* primarily infects the upper small intestine, *E.maxima* colonizes the mid-intestine, and *E. tenella* causes severe lesions in the caeca. This site-specific pathology contributes to impaired nutrient absorption, reduced growth, and increased mortality [3].

To control this problem, farmers often resort to using chemical and ionophore coccidiostats as feed additives; however, their long-term and indiscriminate use has raised public health concerns related to antimicrobial resistance [4]. Immunoprophylaxis also plays an important role in prevention, with vaccines ranging from live virulent strains to embryo-adapted attenuated vaccines and novel subunit formulations [5]. Vaccination can induce protective immunity and reduce reliance on anticoccidial drugs; nevertheless, its increased cost, susceptibility to secondary bacterial enteritis, and challenges in consistent application keep the anticoccidial drugs as the primary industry solution [6].

In response to these challenges, international and regional strategies, such as the European Union’s Farm to Fork initiative, supported by FAO, WOAH, WHO, and UNEP, aim to reduce antimicrobial sales for farm animals by 50% from 2018 levels by 2030 [7]. In parallel, consumers are increasingly concerned about antibiotic use in food animals and are willing to spend more on products labeled as antibiotic-free or with reduced antibiotic use that also minimize ecological footprints. Together, these regulatory and market pressures highlight the need for natural, safe, and one-health-aligned alternatives to conventional anticoccidials [8].

Among the most widely studied natural options are phytochemicals such as polyphenols and flavonoids, which exert anticoccidial effects through immune modulation, enhancement of the gut barrier function, and their antimicrobial and antioxidant properties, thereby improving overall host health [9]. Increasing interest has focused on plant-derived compounds, including oregano [10], garlic [11], neem [12], aloe [13], and turmeric [14], due to their beneficial bioactive compounds [15].

Sugarcane extracts have also been investigated for their potential use in animal feed supplementation [16]. Polygain™, a sugarcane extract rich in polyphenols, has been shown to exhibit beneficial in vitro activity against *Eimeria* spp., reducing their levels to a degree comparable to classical chemical anticoccidials [17]. Furthermore, Polygain™ possesses antioxidant and anti-inflammatory properties, owing to its high polyphenol content, suggesting that it may offer additional beneficial effects in reducing oxidative stress and enhancing immune response in animals [18,19]. However, key knowledge gaps remain, including the limited number of in vivo studies, unclear optimal dosage, and the need for direct comparisons with more common anticoccidials under controlled challenge conditions [20].

Therefore, the present study evaluated the effects of three dietary doses of Polygain™ on growth performance, lesion severity, and oocyst shedding in broiler chickens experimentally challenged with *Eimeria* spp. and compared its efficacy with that of a conventional anticoccidial drug. The objective was to determine whether Polygain™ can serve as a natural alternative to synthetic anticoccidial in broiler production.

## 2. Materials and Methods

### 2.1. Coccidia Preparation

Fecal and caecal content were collected from broiler farms in various provinces in eastern Thailand. *Eimeria* oocysts were purified by saturated sodium chloride flotation and sporulated using standard procedures [21]. Coccidia-free 14-day-old chickens were orally inoculated with the purified oocysts. Approximately 100 oocysts of each *Eimeria* species (*E. acervulina*, *E. maxima*, and *E. tenella*) were inoculated per chicken. At 7 days post-inoculation, the intestinal contents collected for the study included the upper intestinal, middle intestinal, and cecal contents, which were gathered separately and incubated aerobically for four days at room temperature to allow sporulation.

Sporulated oocysts were preserved in 2.5% (*w*/*v*) potassium dichromate at 4°; they were used in these studies within one month after sporulation. Oocyst counts were performed using a McMaster chamber under light microscopy(Chalex Corporation, Wallowa, OR, USA). Total genomic DNA was extracted from purified oocysts using standard methods. The internal transcribed spacer 1 (ITS1) region of *Eimeria* spp. was amplified by PCR following the protocol described by Haug et al. (2007) [22]. This was performed to verify the identity of a single species of the oocyst. After washing, the three *Eimeria* species were propagated via passage through coccidia-free broilers, utilizing sterilized equipment and autoclaved materials to minimize the risk of contamination [22]. All three strains (*E. tenella*, *E. acervulina*, and *E. maxima*) underwent pathogenicity testing following a protocol modified from Tongkamsai et al., 2025 [23].

### 2.2. Compound Preparation

The polyphenol-rich sugarcane extract (PRSE), Polygain™, used in this study was produced by The Product Makers, Melbourne, Australia, using a patented process derived from *Saccharum officinarum* (sugarcane). Gas chromatography–mass spectrometry (GC-MS) untargeted profiling revealed a total of 102 metabolites in the Polygain™ extract; of these, 68 were identified and included 14 amino acids, 34 organic acids, 11 sugars, 5 sugar alcohols, 1 sugar phosphate, and 3 additional compounds [17].

According to the provided specification, the product contains a total polyphenol content of 53,960 mg/L at the starting inclusion level. The following polyphenols and flavonoids have been identified in Polygain™: chlorogenic acid, caffeic acid, sinapic acid, syringic acid, vanillin, homoorientin, orientin, vitexin, swertisin, diosmin, apigenin, tricin, and diosmetin. LC-MS analysis allowed the tentative identification of 7 apigenin-C-glycosides, 3 methoxyluteolin-C-glycosides, and 3 tricin-O-glycosides [18].

### 2.3. Birds and Housing

A total of 144 one-day-old Ross 308 broiler chicks were obtained from the Charoen Pokphand (CP) Group (Bangkok, Thailand). Birds were selected by uniform initial weight upon arrival to ensure consistency across treatment groups and were randomly assigned to six treatment groups with equal average body weights.

Prior to the experiment, a sample size was determined using G.power 3.1.9.7 software to determine the minimum total sample size required for detecting treatment effects with adequate statistical power. The analysis indicated that at least 112 birds were needed to reach adequate power; therefore, the use of 144 was chosen. Each treatment consisted of two replicates of 12 birds.

Prior to bird placement, all housing facilities were cleaned, fumigated with ammonia solution, and flame sterilized. Birds were individually identified using leg bands and maintained under high biosecurity conditions from day 0. All personnel handling the animals wore personal protective equipment. Feeders and drinkers were autoclaved daily. Birds were housed in an isolated, animal-free area within the Department of Veterinary Medicine, RMUTTO.

Each pen measured 2.0 × 1.0 m and was equipped with wire flooring. Birds were provided ad libitum access to filtered drinking water and a commercial basal diet (Top Feed Mills Co., Ltd., Pathum Thani, Thailand). The minimum amounts for each ingredient are as follows: protein, 17%; and fat, 4%. The ambient temperature within each pen was maintained between 33 and 37 °C using infrared heating lamps, and red light was provided for 24 h during the experimental period.

### 2.4. Treatments

Six dietary treatment groups were established. The negative control group (T1-NegCntr) received the basal diet only, remained unchallenged, and received an equivalent volume of distilled water by oral gavage. The positive control group (T2-PosCntr) received a basal diet and was orally challenged with a standardized suspension of sporulated *Eimeria* oocysts on day 14. A drug control group (T3-drug) received the basal diet supplemented with a combination of nicarbazin and narasin. Three experimental groups were provided the basal diet supplemented with Polygain™ at 250 ppm (T4-Polygain250), 500 ppm (T5-Polygain500), and 1000 ppm (T6-Polygain1000) and were challenged with *Eimeria* on day 14.

### 2.5. Eimeria Challenge

On day 14, birds in the positive control and all treatment groups (excluding the negative control) were orally inoculated with a mixed suspension of sporulated *Eimeria* oocysts. Each bird received a single 1 mL dose containing 10,000 oocysts of *Eimeria tenella*, 100,000 oocysts of *Eimeria maxima*, and 200,000 oocysts of *Eimeria acervulina*.

Prior to inoculation, fecal samples were collected from randomly selected birds in each group to confirm the absence of *Eimeria* contamination or early infection using microscopy.

### 2.6. Data Collection

Growth performance parameters, including body weight, were recorded at days 1, 21, and 35 individually for each bird; body weight gain was calculated at 7 days post-infection and at 21 days post-infection. Mortality was monitored daily and expressed as a percentage of the total birds, while survival was reported as the complement.

At 7 and 14 days post-infection (dpi; days 28 and 35 of age, respectively), six birds per treatment were randomly selected and humanely euthanized for postmortem evaluation. Fecal samples were collected to determine *Eimeria* oocyst shedding using the McMaster counting technique. Intestinal lesion scoring was conducted in accordance with the method described by Johnson and Reid [24]. Based on collected data, the following indices were calculated: oocyst index, anticoccidial index (ACI) [25], growth/survival ratio (GSR), relative lesion score, and percentage optimum anticoccidial activity (POAA) [26].

### 2.7. Percent Optimum Anticoccidial Activity (POAA)

POAA is an index used to evaluate the efficacy of anticoccidial treatments by integrating both growth and survival parameters. The growth and survival ratio (GSR) was calculated for each treatment group using the following formula:

$$Growth\;and\;survival\;ratio\left(GSR\right)=\frac{Final\;pen\;weight\;}{Initial\;pen\;weight\;}$$

This ratio reflects the overall growth performance and survivability of the group relative to its status at the time of *Eimeria* challenge. The POAA was then calculated using the formula described by Conway and McKenzie [27].

$$Percentage\;optimum\;Anticoccidial\;Activity\;\left(POAA\right)=\frac{\left(GSR\;of\;an\;infected\;group-GSR\;of\;infected\;controls\right)}{\left(GSR\;of\;noninfected\;medicated\;controls-GSRof\;infected\;controls\right)}\times 100$$

A POAA value > 50% was considered indicative of a sensitive response, while values ≤ 50% were classified as resistant.

### 2.8. Reduction Lesion Score (RLS)

At necropsy, intestinal lesions associated with coccidiosis were assessed using the method described by Johnson and Reid [24]. The total mean lesion score was calculated as the sum of the average lesion scores for *Eimeria tenella*, *E. acervulina*, and *E. maxima*. Lesions were scored individually for each bird on a scale from 0 (no lesions) to 4 (severe lesions). Birds that died prior to scoring were assigned a lesion score of 4 [28]. For each medicated treatment group, the reduction in lesion score (RLS) was calculated using the following formula:

$$RLS=\frac{\left(average\;lesion\;score\;in\;infected\;unmedicated\;group-average\;lesion\;score\;in\;medicated\;group\right)}{average\;lesion\;score\;in\;infected\;unmedicated\;group}\times 100$$

An RLS value > 50% was interpreted as indicative of a sensitive response to the treatment, whereas a value ≤ 50% was considered resistant.

### 2.9. Oocyst per Gram (OPG)

To assess *Eimeria* replication, the number of oocysts per gram (OPG) of feces was determined at 6 and 20 days post-infection (dpi). Chickens were temporarily removed from their pens and transferred to sterile, single-use containers to facilitate the collection of fecal samples. A single dropping was collected from each of the 12 birds within each treatment group, and the individual birds were weighed separately. In total, twelve fecal samples were obtained from each treatment group for subsequent analysis.

Oocyst counts were performed using the McMaster technique [29] under a compound microscope (Nikon E-400, Nikon Corporation, Tokyo, Japan). at 10× magnification. Each sample was counted in triplicate, and results were reported as the mean number of oocysts per gram of feces (OPG) [30].

### 2.10. Relative Oocyst Production (ROP)

Relative oocyst production (ROP) was calculated to evaluate the effect of treatments on *Eimeria* replication. The ROP (%) was determined using the following formula:

$$ROP\left(\%\right)=\frac{\left(avergae\;oocyst\;output\;in\;treated\;infected\;group\right)}{\left(average\;oocyst\;output\;in\;infected\;unmedicated\;group\right)}\times 100$$

This parameter was used to classify the sensitivity of *Eimeria* spp. to treatment. An ROP value greater than 15% was interpreted as resistant, while an ROP value less than 15% was sensitive [31].

### 2.11. The Anticoccidial Index (ACI)

The anticoccidial index (ACI) was calculated to assess the overall efficacy of the anticoccidial treatments. It combines performance, lesion, and oocyst parameters into a single index using the following formula:

$$Anticoccidial\;index=\left(relative\;weight\;gain\;rate+survival\;rate\right)-\left(lesion\;score+oocyst\;value\right)$$

The oocyst index was determined using this formula:

$$Oocyst\;index=\left(\frac{OPG\;in\;each\;group}{OPG\;in\;positive\;control}\right)\times 100$$

An ACI higher than 180 indicated excellent activity, from 160 to 179 indicated moderate activity, from 120 to 159 indicated limited activity, and a value lower than 120 indicated inefficacy [32].

### 2.12. Statistical Analysis

Data were analyzed in a completely randomized design with pen means serving as the experimental unit. Growth performance was assessed using analysis of variance (ANOVA). Assumptions of ANOVA were checked using the Shapiro–Wilk test for normality and Levene’s test for homogeneity of variances. Differences among treatments were determined using Tukey’s Honest Significant Difference (HSD) test, with statistical significance declared at *p* < 0.05.

Lesion scores and oocyst per gram (OPG) counts were analyzed using the non-parametric Kruskal–Wallis test, followed by Dwass–Steel–Critchlow–Flinger pairwise comparisons. Group differences in mortality were assessed using a Chi-square test of independence.

All statistical analyses were conducted using GraphPad Prism v18 (GraphPad Software, San Diego, CA, USA). Results are presented as mean ± standard deviation (SD), and treatments with different superscripts differ significantly at *p* < 0.05.

### 2.13. Safety and Ethical Considerations

All procedures involving animals were conducted in accordance with the guidelines of the Institutional Animal Care and Use Committee (IACUC) and were approved under protocol number RMUTTO-ACUC-2-2025-008, and adhered to the ARRIVE guidelines to ensure ethical standards in animal research. Appropriate personal protective equipment (PPE) was used during the handling of *Eimeria* oocysts to minimize contamination risks, following established biosecurity protocols. All test substances, including nicarbazin and narasin, as well as Polygain™, were handled and administered according to the manufacturers’ guidelines to ensure safety and efficacy. Birds were monitored daily for signs of illness, and any adverse health effects were promptly reported and managed in accordance with the approved protocol.

## 3. Results

### 3.1. Body Weight Gain (BWG) and Growth Performance

**Figure 1:**
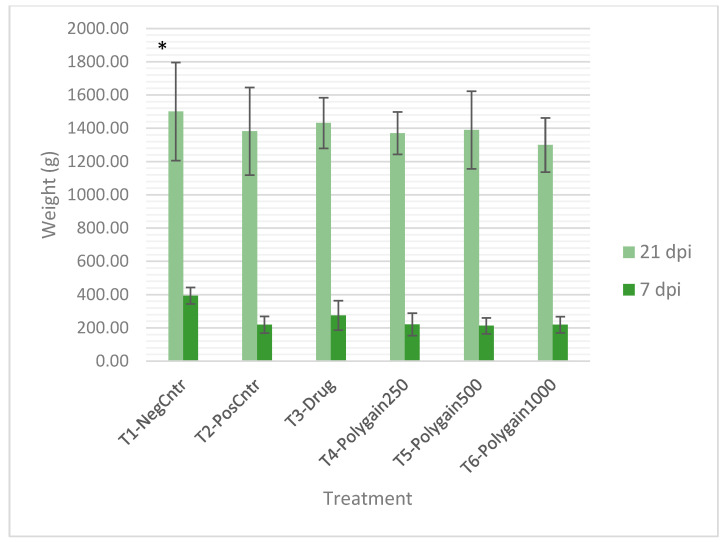
Mean body weight (g) of broilers at 7 and 21 days post-infection (dpi) with *Eimeria* spp. under different dietary treatments. Values are presented as mean ± SD (n = 12 birds per group). T1-NegCntr = uninfected, untreated negative control; T2-PosCntr = infected, untreated positive control; T3-Drug = infected + nicarbazin and narasin; T4-Polygain250, T5-Polygain500, and T6-Polygain1000 = infected + Polygain™ at 250, 500, or 1000 ppm, respectively. (*) indicates significant differences among treatments at each time point (*p* < 0.05).

At 7 days post-infection (dpi), birds in the uninfected, untreated control group (T1) exhibited significantly higher body weight gain (BWG) (393.67 ± 49.72 g) compared to all infected groups (T2–T6), which displayed weight reductions ranging from 119 to 181 g (*p* < 0.05). This supports the acute growth-suppressive effects of *Eimeria* infection. (Figure 1).

By 21 dpi, no significant differences in BWG were observed among treatment groups (*p* > 0.05; Figure 1), indicating compensatory growth in infected birds. Inclusion of Polygain™ did not negatively affect BWG, with values comparable to the positive control, suggesting that Polygain™ did not impair growth.

The final pen weight and growth/survival ratio (GSR) reflected these trends. T1 achieved the highest final pen weight (10,191.00 ± 93.72 g) and GSR (1.86), while all infected groups ranged from 1.46 to 1.59. Among infected groups, the drug treatment (T3) achieved the highest GSR (1.59), whereas Polygain™ groups showed values comparable to the positive control (1.46–1.47). Percent optimum anticoccidial activity (POAA) was highest in T3 (31.80%). Polygain™ treatments exhibited lower POAA values (0.98–4.09%), maintaining resistance under the current challenge model (Table 1).

### 3.2. Lesion Scores

Lesion scores in the upper-, mid-, and caecum-intestinal regions, as well as the total lesion score (TLS), are presented in Table 2. The negative control (T1-NegCntr) showed no detectable lesion in any intestinal segment at either 7 or 21 dpi. In contrast, the infected positive control (T2-PosCntr) exhibited the highest lesion scores at 7 dpi (U: 1.9 ± 0.9, M: 1.4 ± 1.8, C: 2.9 ± 0.9), resulting in the highest TLS (6.3 ± 2.7), confirming successful infection. All treated groups (T3–T6) demonstrated significantly lower TLS compared to T2 (*p* < 0.05).

By 21 dpi, lesion scores decreased in all groups. T2 retained higher scores in the upper- and mid-intestine, while T6 (Polygain™ 1000) showed complete resolution in the caecum (lesion score = 0). A dose–response trend was observed in the Polygain™ groups, particularly in the upper and caecal regions, indicating increasing protection with dose.

**Figure 2:**
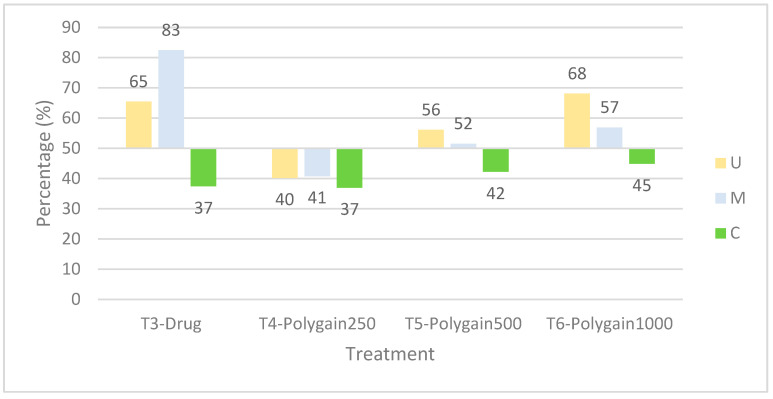
Percentage reduction in lesion scores by intestinal region at 7 days post-infection (dpi). Reductions were calculated for the upper intestine (U), mid-intestine (M), and caecum (C). Data represent mean values per treatment group, T3 = nicarbazin + narasin, T4 = Polygain™ 250 ppm, T5 = Polygain™ 500 ppm, T6 = Polygain™ 1000 ppm). The dashed line at 50% reduction indicates the threshold used to classify *Eimeria* sensitivity; values > 50% indicate a sensitive response, whereas values ≤ 50% indicate resistance to the treatment.

Percentage reduction in lesion scores (RLS) further supported these observations (Figure 2). T3, T5, and T6 showed protective responses in the upper intestine at 7 dpi. In the mid-intestine, T3 was most protective, while T5 and T6 also reduced damage. No significant protection was observed in the caecum at 7 dpi.

### 3.3. Oocyst per Gram and Relative Oocyst Production

Oocyst per gram (OPG) counts at 6 and 20 dpi are shown in Table 3 At 6 dpi, T2 exhibited the highest shedding (15,264,608 OPG). T3 significantly reduced OPG to 435,308, while Polygain™ at 250, 500, and 1000 ppm yielded 254,667, 479,917, and 476,408 OPG, respectively. No significant differences were found among treated groups at this time point (Table 3).

By 20 dpi, oocyst shedding was markedly reduced in all groups. T3 again had the lowest OPG (79), followed by Polygain™ 250 (407), 500 (472), and 1000 (914). Among the Polygain™ groups, the 250 ppm dose produced the most favorable oocyst profile at 20 dpi.

Relative oocyst production (ROP) values were markedly reduced across all medicated groups. The standard drug group (T3) exhibited an ROP of 2.8%, indicating sensitivity according to the 15% threshold. Polygain™ treatments also demonstrated low ROP values: 1.6%, 3.1%, and 3.1% for T4, T5, and T6, respectively. All ROP values remained well below the 15% resistance cutoff, confirming the sensitivity of *Eimeria* spp. to both the conventional drug and the tested doses of Polygain™.

### 3.4. Anticoccidial Index

The anticoccidial index (ACI), which integrates relative body weight gain rates (rBWGR), survival, lesion scores, and oocyst output, is presented in Table 4. T2 (infected–untreated) showed the lowest rBWGR (55.63) and survival (91.66%), highlighting the impact of *Eimeria* infection. All treated groups maintained high survival (95.83%). Mortality ranged from 0 in the negative control to 13.3% in the positive control; there was no significant difference among treatments.

T3- Drug achieved the highest ACI (160.13), classified as moderate efficacy. Polygain™ treatments yielded ACI scores of 143.46–146.54, reflecting limited efficacy. Increasing Polygain™ dose from 250 to 1000 ppm did not lead to meaningful improvements in ACI, suggesting a plateau effect. Nonetheless, although slightly lower relative body weight gain rates (rBWGRs) were observed in the Polygain™ groups, the lesion score for Polygain™ at 1000 ppm was nearly equivalent to that of the drug-treated group (Table 4). Importantly, none of the treatments negatively affected survival or overall performance.

## 4. Discussion

The most significant limitation to the efficacy of anticoccidial drugs is the development of drug-resistant *Eimeria* strains, which have arisen from the continuous use of these medications, thereby raising substantial concerns and leading to regulatory restrictions. This study evaluated the anticoccidial efficacy of Polygain™, a sugarcane-derived polyphenol feed material, in broiler chickens experimentally challenged with *Eimeria* spp. The results demonstrate that Polygain™ mitigated the impact of coccidial infection, as evidenced by reductions in oocyst shedding and lesion severity. Although body weight recovery in Polygain groups was comparable to the infected untreated control, supplementation did not exacerbate growth impairment under challenge conditions.

At 7 days post-infection (dpi), birds in the Polygain™ as well as nicarbazin and narasin groups exhibited lower BWG compared to the uninfected controls, reflecting the acute impact of high-dose *Eimeria* infection [33]. However, by 21 dpi, both treatment groups showed substantial recovery, with BWG approaching or exceeding those of the unmedicated-challenged group.

Most notably, Polygain™ achieved a >99% reduction in oocyst, comparable to the suppression observed with nicarbazin and narasin. At 6 dpi, oocyst counts in the Polygain™ group were similar to those in the drug-treated group. Remarkably, these values were lower than those reported for other natural compounds such as silymarin, quercetin, resveratrol, or nerolidol [34], highlighting the potency of Polygain™ in reducing *Eimeria* replication.

Relative oocyst production (ROP) values further confirmed this effect, with all Polygain™ groups exhibiting ROP values well below the 15% resistance threshold. Specifically, ROP values for the drug, Polygain250, Polygain500, and Polygain1000 groups were 2.8%, 1.6%, 3.1%, and 3.1%, respectively, indicating *Eimeria* sensitivity across all treatments [31].

Another important observation was the low mortality rate (<5%) in the Polygain™ group. This was notably lower than values reported for other natural compounds, such as garlic (12.5%), moringa (5%), and oregano essential oil (12.5%) [35]. This supports the safety and robustness of Polygain™ as a potential anticoccidial feed material.

Lesion scores, particularly in the mid-intestine and cecum, were also significantly reduced in the Polygain™ group compared to unmedicated-challenged birds. Given that *E. acervulina* and *E. tenella* preferentially infect the upper intestine and cecum, respectively [36,37], these findings may indicate targeted protection against these species. Notably, one Polygain™ treatment group showed complete remission of caecal lesions by 20 dpi. This effect may be mediated by microbial-derived polyphenol metabolites, which are primarily generated in the ceca and colon, where polyphenols exhibit high bioavailability [38]. Similar observations have been made with essential oils, which enhance villus length in the duodenum and ceca but have limited impact on the jejunum or ileum [39]. Nevertheless, it is important to acknowledge that a reduction in 20 dpi could also reflect the host-adaptive immune response [40], or a decreased presence of infectious oocyst in the environment [41], factors that may contribute alongside the effects of the polyphenol supplementation [42].

Despite these results, the calculated anticoccidial index (ACI) for the three Polygain™ treatments fell below the ≥160 threshold, and is considered a limited efficacy [31]. This suggests that while Polygain™ demonstrates notable effects, it may not yet serve as a stand-alone replacement for synthetic drugs in severe infections. Furthermore, the absence of a dose-dependent increase in the ACI suggests a plateau effect. This may be related to the limited intestinal bioavailability of polyphenols [38] or their preferential utilization, mitigating oxidative stress during infection, rather than being directly solely toward inhibiting parasite replication [43,44].

The mechanism of action for Polygain™ likely involves multiple, complementary pathways. Polyphenols are well-documented for their antioxidant and anti-inflammatory properties, as well as their ability to modulate the gut microbiome, all of which may enhance host resilience to enteric infections [45]. These effects may begin in early life, supporting intestinal epithelial development, protecting against *Eimeria* spp.-induced damage, and promoting mucosal recovery [43].

Polyphenols in Polygain™, as chlorogenic acid, have also been shown to penetrate cellular membranes and exert potent intracellular antioxidant activity in vitro and in poultry. Specifically, it activates the nuclear factor erythroid 2–related factor 2 (Nrf2) signaling pathway [18,46,47], which stimulates the production of endogenous antioxidant enzymes. This mechanism may disrupt redox balance within *Eimeria* sporozoites, destabilizing their membranes and contributing to parasite death [48]. Concurrently, the antioxidant effects may help the host mitigate oxidative stress associated with coccidia infection, a process that compromises the intestinal barrier, reduces feed intake and nutrient absorption, and ultimately impairs growth performance [44].

From a practical standpoint, Polygain™ may serve as a supportive component of integrated coccidiosis control programs. It could be particularly valuable in systems aiming to reduce or eliminate synthetic anticoccidials and may be used as a starter supplement or in combination with probiotics or other phytogens to promote gut microbiota resilience, as demonstrated for similar feed materials [43]. Moreover, feed materials such as Polygain™ have been explored in early-life nutritional programming in other species [49], suggesting potential for cross-generational immunomodulation, a field of growing relevance in the poultry industry [50,51]. Pre- and probiotics have also been shown to induce beneficial microbiological and immunological changes in the gastrointestinal tract without adverse effects, thereby supporting immune functionality in young birds [52].

Importantly, the development of such systems is critical in the face of increasing antimicrobial resistance (AMR), which poses a major threat to public health. In 2021, AMR was associated with an estimated 41.71 million deaths globally [53], prompting its designation as a top priority in the WHO’s global action plan [54].

Environmental concerns also warrant reduced use of synthetic coccidiostats like nicarbazin and narasin. Risk assessments based on European poultry production estimate risk quotients as high as 28 under worst-case scenarios, indicating high environmental hazard. Ionophores may accumulate in lipophilic compartments such as soil and sediments, where long-term ecotoxicological effects remain largely unassessed [55].

While these findings highlight the potential of Polygain™ as a sustainable and safe alternative to conventional anticoccidials, several limitations of this exploratory study should be acknowledged. The relatively small sample size (n = 12 per group) may restrict the generalizability of the results, and only one broiler strain (Ross 308) was evaluated; therefore, responses in other breeds of slower-growing strains [56] or geographical location may differ [57]. In addition, the mixed *Eimeria* spp. challenge model may not fully reflect field conditions, where pathogen loads and species composition are more variable [58]. The study duration, limited to 21 days post-infection, also limits the assessment of longer-term outcomes, knowing that the recovery phase can take up to 30 DPI [59]. Finally, although efficacy was classified as limited, Polygain™ remains cost-competitive with current options, which strengthens its potential for adoption if efficacy is improved.

Broilers have a life span of just 35 to 45 days and are exposed to ionophores throughout their production cycle. Despite rapid metabolism and a minimum 6-day withdrawal period, ionophores can still disrupt poultry supply chains [60]. Moreover, these compounds pose risks of cross-contamination, especially in layer systems, where nicarbazin has been associated with reduced egg weight, hatchability, and shell pigmentation [61]. While prohibited in layers due to these risks, residues may still be detected due to feed mill cross-contamination [62]. In contrast, Polygain™, as a natural feed material with no adverse effects reported in layers, presents a safer, innovative, and more sustainable alternative for contemporary poultry production systems [63].

## 5. Conclusions

Polygain™ reduced oocyst output and intestinal lesions, highlighting its potential as a natural and safe supplement in poultry systems aiming to transition away from antibiotics. However, its anticoccidial index (ACI) classified the supplement as limited, indicating that it may not fully replace conventional drugs at this stage. While no statistically significant difference was detected between the tested inclusion levels in this study, future research should investigate its performance in large-scale commercial trials and long-term assessments across poultry classes (turkeys, breeders, and layers) are required. Its role in early-life nutritional programming to promote cross-generational immunomodulation also merits further exploration.

## Figures and Tables

**Table 1:** Effects of dietary treatments on broiler growth survival rate (GSR) and percent optimum anticoccidial activity (POAA) at 7 and 21 days post-infection (dpi) with *Eimeria* spp.

Treatment	GSR	POAA
T1-NegCntr	1.86	N.A
T2-PosCntr	1.46	N.A
T3-Drug	1.59	31.80
T4-Polygain250	1.47	4.09
T5-Polygain500	1.46	0.98
T6-Polygain1000	1.46	1.68

Values are presented as mean ± standard deviation (n = 12 per group). T1 = uninfected–untreated negative control; T2 = infected–untreated positive control; T3 = infected + nicarbazin and narasin; T4–T6 = infected + Polygain™ at 250, 500, and 1000 ppm, respectively. GSR = growth survival rate (Final PW/Initial PW); POAA = percent optimum anticoccidial activity. N.A = not applicable or not assessed for that treatment

**Table 2:** Lesion scores in the upper (U), mid (M), and caecal (C) intestinal regions at 7 and 21 days post-infection (dpi) with *Eimeria* spp.

Treatment	Lesion Scores							
	7 dpi				21 dpi			
	U	M	C	TLS	U	M	C	TLS
T1-NegCntr	0 ^A^	0 ^A^	0 ^A^	0 ^A^	0 ^A^	0 ^A^	0 ^A^	0 ^A^
T2-PosCntr	1.9 ± 0.9 ^B^	1.4 ± 1.8 ^B^	2.9 ± 0.9 ^B^	6.3 ± 2.7 ^B^	1.7 ± 0.6 ^B^	1.3 ± 0.6 ^B^	0 ^A^	3.0 ± 1.1 ^B^
T3-Drug	0.6 ± 0.4 ^C^	0.2 ± 0.4 ^C^	1.8 ± 1.0 ^C^	2.8 ± 0.8 ^BC^	0.6 ± 1.5 ^CD^	0.5 ± 1.1 ^CD^	0.3 ± 1.1 ^B^	1.6 ± 0.8 ^AB^
T4-Polygain250	1.1 ± 1.0 ^BC^	0.8 ± 1.0 ^B^	1.8 ± 1.2 ^C^	3.9 ± 0.5 ^C^	0.9 ± 0.8 ^CD^	0.7 ± 0.6 ^CD^	0.1 ± 0.3 ^AB^	1.7 ± 0.4 ^B^
T5-Polygain500	0.8 ± 1.0 ^C^	0.6 ± 1.1 ^B^	1.6 ± 0.9 ^C^	3.2 ± 0.5 ^C^	1.0 ± 0.7 ^D^	0.7 ± 0.6 ^CD^	0.3 ± 0.6 ^B^	2.1 ± 0.3 ^B^
T6-Polygain1000	0.6 ± 1.1 ^C^	0.6 ± 1.1 ^B^	1.6 ± 0.9 ^C^	2.9 ± 06 ^C^	0.5 ± 0.6 ^C^	0.4 ± 0.6 ^C^	0 ^A^	1.0 ± 0.3 ^AB^

Mean values are expressed as mean ± standard deviation (n = 12 per group). U = upper intestine; M = mid-intestine; C = caecum; TLS = total lesion score. Different superscript letters within the same column indicate statistically significant differences (*p* < 0.05).

**Table 3:** Oocyst shedding and reduction in oocyst output at 6 and 20 days post-infection (dpi) in broilers treated with anticoccidial agents.

Treatment	Oocyst Per Gram	
	6 dpi	20 dpi
NegCntr, T1	0 ^a^	0 ^A^
PosCntr, T2	15,264,608 ^b^	777 ^B^
T3, drug	435,308 ^c^	79 ^B^
T4, Polygain™250	254,667 ^c^	407 ^B^
T5, Polygain™500	479,917 ^c^	472 ^B^
T6, Polygain™1000	476,408 ^c^	914 ^B^

Values represent the mean oocyst output (oocysts per gram of feces) for each treatment group at 6 and 20 days post-infection (dpi). Different superscript letters (a–c) indicate statistically significant differences (*p* < 0.05) in OPG at 6 dpi. While uppercase letter (A–B) indicate significant differences (*p* < 0.05) in PG at 20 dpi. T1 = uninfected-untreated control; T2 = infected-untreated control; T3 = nicarbazin and narasin; T4–T6 = Polygain™ at 250, 500, and 1000 ppm, respectively.

**Table 4:** Anticoccidial Index (ACI) and its components in broilers treated with anticoccidial agents at 7 days post-infection.

Treatment	rBWGR	Mortality %	Survival Rate (%)	Lesion Score	ROP	ACI
T1-NegCntr	N.A	0.00% ^A^	100	N.A	N.A	N.A
T2-PosCntr	55.63	13.30% ^A^	91.66	N.A	N.A	N.A
T3-Drug	69.90	8.30% ^A^	95.83	2.75 ^AB^	2.85	160.13
T4-Polygain250	56.22	7.70% ^A^	95.83	3.85 ^B^	1.67	146.54
T5-Polygain500	54.00	7.70% ^A^	95.83	3.23 ^B^	3.14	143.46
T6-Polygain1000	55.67	7.70% ^A^	95.83	2.85 ^B^	3.12	145.54

rBWGR = Relative body weight gain rate; calculated relative to the uninfected-untreated group. Survival rate = percentage of birds that survived to 7 dpi. ROP = relative oocyst production expressed as a percentage of the infected-untreated control. ACI = Anticoccidial Index, ACI > 180 = excellent; 160–179 = moderate; 120–159 = limited; <120 = ineffective. N.A = not applicable or not assessed for that treatment. Values within a column with different superscripts (A, B) differ significantly at *p* < 0.05.

## Data Availability

Data will be made available on request.

## Abbreviations

The following abbreviations are used in this manuscript:

ROP	Relative Oocyst Production
RLS	Reduction Lesion Score
POAA	Percent Optimal Anticoccidial Activity
ACI	Anticoccidial Index
PRSE	Polyphenol-Rich Sugarcane Extract
GC-MS	Gas Chromatography Mass Spectrometer
CP	Charoen Pokphand
FCR	Feed Conversion Ratio
GSR	Growth Survival Ratio
OPG	Oocyst per Gram
ANOVA	Analysis of Variance
CRD	Completely Randomized Design
IACUC	Institutional Animal Care and Use Committee
PPE	Protective Personal Equipment
DPI	Day Post-Infection
BWG	Body Weight Gain
U	Upper Intestine
M	Mid-Intestine
C	Caecum
Nrf2	Nuclear Factor Erythroid–Related Factor 2
AMR	Antimicrobial Multiple Resistance
